# Control of *hmu* Heme Uptake Genes in *Yersinia pseudotuberculosis* in Response to Iron Sources

**DOI:** 10.3389/fcimb.2018.00047

**Published:** 2018-02-22

**Authors:** Leah Schwiesow, Erin Mettert, Yahan Wei, Halie K. Miller, Natalia G. Herrera, David Balderas, Patricia J. Kiley, Victoria Auerbuch

**Affiliations:** ^1^Department of Molecular, Cell, and Developmental Biology, University of California, Santa Cruz, Santa Cruz, CA, United States; ^2^Department of Biomolecular Chemistry, University of Wisconsin-Madison, Madison, WI, United States; ^3^Department of Microbiology and Environmental Toxicology, University of California, Santa Cruz, Santa Cruz, CA, United States

**Keywords:** *Yersinia*, IscR, Hmu, heme uptake, blood

## Abstract

Despite the mammalian host actively sequestering iron to limit pathogenicity, heme (or hemin when oxidized) and hemoproteins serve as important sources of iron for many bloodborne pathogens. The HmuRSTUV hemin uptake system allows *Yersinia* species to uptake and utilize hemin and hemoproteins as iron sources. HmuR is a TonB-dependent outer membrane receptor for hemin and hemoproteins. HmuTUV comprise a inner membrane ABC transporter that transports hemin and hemoproteins from the periplasmic space into the bacterial cytoplasm, where it is degraded by HmuS. Here we show that *hmuSTUV* but not *hmuR* are expressed under iron replete conditions, whereas *hmuR* as well as *hmuSTUV* are expressed under iron limiting conditions, suggesting complex transcriptional control. Indeed, expression of *hmuSTUV* in the presence of inorganic iron, but not in the presence of hemin, requires the global regulator IscR acting from a promoter in the intergenic region between *hmuR* and *hmuS*. This effect of IscR appears to be direct by binding a site mapped by DNaseI footprinting. In contrast, expression of *hmuR* under iron limiting conditions requires derepression of the ferric uptake regulator Fur acting from the *hmuR* promoter, as Fur binding upstream of *hmuR* was demonstrated biochemically. Differential expression by both Fur and IscR would facilitate maximal hemin uptake and utilization when iron and heme availability is low while maintaining the capacity for periplasmic removal and cytosolic detoxification of heme under a wider variety of conditions. We also demonstrate that a *Y. pseudotuberculosis* Δ*iscR* mutant has a survival defect when incubated in whole blood, in which iron is sequestered by heme-containing proteins. Surprisingly, this phenotype was independent of the Hmu system, the type III secretion system, complement, and the ability of *Yersinia* to replicate intracellularly. These results suggest that IscR regulates multiple virulence factors important for *Yersinia* survival and growth in mammalian tissues and reveal a surprising complexity of heme uptake expression and function under differing conditions of iron.

## Introduction

The battle for iron between host and pathogen has shaped both mammalian nutritional immunity and microbial pathogenesis (Cassat and Skaar, 2013). Free iron is present in mammalian tissues at an astonishing 10^−24^ M as a result of numerous iron binding proteins. In addition, inflammatory cytokines such as IL-6 induce hypoferremia, further limiting the amount of iron available to invading pathogens. However, microbial pathogens such as the plague agent *Yersinia pestis* and the enteropathogens *Y. pseudotuberculosis* and *Y. enterocolitica* encode numerous iron acquisition systems (Forman et al., 2010). For example, the siderophore yersiniabactin has an extremely high affinity for ferric iron that enables *Yersinia* to remove iron from a number of mammalian proteins (Haag et al., 1993). While the majority of *Yersinia* iron acquisition systems take up inorganic iron, some, such as the Hmu system, take up host-derived heme and heme-containing proteins, important sources of iron for many pathogens (Anzaldi and Skaar, 2010).

The importance of iron acquisition during infection is underscored by evidence suggesting that a correlation exists between highly pathogenic *Yersinia* and their ability to successfully acquire iron *in vivo* (Burrows and Jackson, 1956; Robins-Browne and Prpic, 1985). Indeed, there are at least seven different functional iron acquisition systems (Ybt, Yfe, Yfu, Yiu, Feo, Fet, and Hmu) utilized by human pathogenic *Yersinia* (Perry et al., 2015). The most well-characterized *Yersinia* iron acquisition system is the siderophore yersiniabactin (Ybt), which is required for full virulence in a mouse model of bubonic plague (Bearden et al., 1997). The Yfu and Yiu systems are ABC transporters that play a role in ferric iron uptake under aerobic conditions, while the Feo and Fet systems can uptake ferrous iron under microaerophilic conditions (Perry et al., 2015). Recently, work has shown that the Feo system can also uptake ferric iron (O'Connor et al., 2017). The Yfe system has been shown to play a role in both ferric and ferrous iron transport under aerobic and microaerophillic conditions, respectively (Perry et al., 2015).

Heme and hemoproteins provide the largest source of iron for bacterial pathogens within the host (Choby and Skaar, 2016). The HmuRSTUV (HemRSTUV in *Yersinia enterocolitica*) hemin uptake system has been characterized in *Y. pestis* and *Y. enterocolitica* (Stojiljkovic and Hantke, 1992, 1994; Hornung et al., 1996; Thompson et al., 1999). HmuR is a TonB-dependent outer membrane transporter that is required for the utilization of heme, hemoglobin, hemoglobin-haptoglobin, myoglobin, hemopexin, and heme albumin in *Y. pestis* (Thompson et al., 1999). HmuTUV make up an inner membrane ABC transporter that is required for the uptake of hemin and all hemoproteins except hemopexin and haptoglobin-hemoglobin into the cytoplasm (Thompson et al., 1999). The cytoplasmic protein HmuS has been proposed to play a role in the detoxification and degradation of hemin, as deletion of the HmuS homolog in *Yersinia enterocolitica*, HemS, was reported to be lethal (Stojiljkovic and Hantke, 1994). Consistent with this function, studies in *Yersinia pseudotuberculosis* have shown that HmuS releases iron via degradation of heme to biliverdin in the presence of molecular oxygen, an electron donor such as NADPH, and ferrodoxin-NADP^+^ reductase (Onzuka et al., 2017).

Sensing of low iron environments to regulate virulence programs within the host is a hallmark of bacterial pathogenesis (Skaar, 2010). Accordingly, hemin uptake pathways in several bacterial pathogens, including *Staphylococcus aureus, Neisseria meningitidis*, and *Yersinia pestis*, are regulated by the ferric uptake regulator Fur (Thompson et al., 1999; Choby and Skaar, 2016). Under iron replete conditions, Fur is bound by ferrous iron (Fe^2+^), thereby allowing it to bind target operator sequences (Fur boxes) in order to repress transcription (Troxell and Hassan, 2013). Conversely, as iron becomes limiting, Fur is no longer bound by iron leading to derepression of iron uptake systems (Troxell and Hassan, 2013). *Yersinia pestis hmuR* contains a Fur box in its promoter region and its transcription is responsive to iron availability (Thompson et al., 1999). Expression of *Yersinia enterocolitica hemR* was upregulated in the peritoneal cavity, Peyer's patches, and spleen during infection, suggesting a role for low-iron responsive hemin uptake in these tissues during infection (Jacobi et al., 2001).

Unexpectedly, our group demonstrated that in *Yersinia pseudotuberculosis* the iron-sulfur coordinating transcription factor IscR also regulates *hmuSTUV* expression in addition to the Ysc virulence-associated type III secretion system (T3SS) and a number of other genes (Miller et al., 2014). IscR coordinates a [2Fe-2S] cluster that affects DNA binding specificity to positively or negatively affect transcription of downstream genes (Rajagopalan et al., 2013). Holo-IscR binds so called type I and II motifs while apo-IscR can only bind type II motifs (Nesbit et al., 2009; Rajagopalan et al., 2013). In *E. coli*, IscR has been shown to respond to oxidative stress and oxygen limitation as well as iron limitation, suggesting that oxygen, as well as iron, may be important in the regulation of hemin uptake in *Yersinia* species (Giel et al., 2006; Yeo et al., 2006; Wu and Outten, 2009).

In this study, we examine how expression of *hmuRSTUV* is controlled by iron sources and dissect the contribution of at least two global regulators, Fur and IscR, to *hmu* expression. We demonstrate that IscR but not Fur binds to an intergenic region between *hmuR* and *hmuS* and potentiates *hmuSTUV* expression. Furthermore, we examine the role of IscR in survival in whole blood, a rich source of heme. Although, survival in whole blood did not require the Hmu system, it did require IscR. This work increases our understanding of iron control of heme uptake and the role of IscR in *Yersinia pseudotuberculosis* survival and growth in mammalian tissues.

## Materials and methods

### Bacterial strains, plasmids, and growth conditions

All strains used in this study are listed in Table 1. *Y. pseudotuberculosis* strains were grown in either 2xYT or M9 minimal media supplemented with casamino acids referred to here as M9, at 26°C with shaking at 250 rpm, unless otherwise indicated (Cheng et al., 1997).

**Table 1:** Bacterial strains used in this study.

**Strain**	**Background**	**Mutation(s)**	**Reference**
*E. coli*	K12		MG1655
*Y. pseudotuberculosis* WT	IP2666	Naturally lacks full length YopT	Bliska et al., 1991
Δ*iscR*	IP2666	Δ*iscR*	Miller et al., 2014
pYV^−^	IP2666	Δ*yscBL* pYV cured	Auerbuch et al., 2009
ΔpYV^−^/Δ*iscR*	IP2666	Δ*iscR* plasmid cured	This study
Δ*yscNU*	IP2666	Δ*yscNU*	Balada-Llasat and Mecsas, 2006
Δ*phoP*	IP2666	Δ*phoP*	Miller et al., 2016
Δ pYV^−^/*ΔhmuRSTUV*	IP2666	Δ*hmuRSTUV* plasmid cured	This study
Δ pYV-/Δ*hmuSTUV*	IP2666	Δ*hmuSTUV* plasmid cured	This study
Δ*hmuRSTUV*	IP2666	Δ*hmuRSTUV*	This study
Δ*hmuSTUV*	IP2666	Δ*hmuSTUV*	This study

### Construction of *Y. pseudotuberculosis* mutant strains

The Δ*hmuRSTUV* and Δ*hmuSTUV* mutants were generated via splicing by overlap extension (Warrens et al., 1997). Primer pairs F5/R5Δ*hmuRSTUV* and F5/R5Δ*hmuSTUV* (Table 2) were used to amplify ~1,000 bp 5′ of *hmuRSTUV* and *hmuSTUV*, respectively. Primer pair F3/R3Δ*hmuRSTUV* were used to amplify ~1,000 bp 3′ of Δ*hmuRSTUV* (Table 2). Amplified PCR fragments served as templates in an overlap extension PCR using the outside primers F5/R3Δ*hmuRSTUV* and F5Δ*hmuSTUV*/R3Δ*hmuRSTUV* for Δ*hmuRSTUV* and Δ*hmuSTUV*, respectively. The resulting ~2 kb fragments were cloned into the TOPO TA cloning vector (Invitrogen) and further subcloned into a *Bam*HI- and *Not*I-digested pSR47s suicide plasmid (λpir-dependent replicon, kanamycin^R^ (Kan^R^), *sacB* gene conferring sucrose sensitivity) (Merriam et al., 1997; Andrews et al., 1998). Recombinant plasmids were transformed into *E. coli* S17-1 λpir competent cells and later introduced into *Y. pseudotuberculosis* IP2666 via conjugation. The resulting Kan^R^, irgansan^R^ (*Yersinia* selective antibiotic) integrants were grown in the absence of antibiotics and plated on sucrose-containing media to select for clones that had lost *sacB* (and by inference, the linked plasmid DNA). Kan^S^, sucrose^R^, congo red-positive colonies were screened by PCR and sequencing.

**Table 2:** *Y. pseudotuberculosis* primers used in this study.

**Name**	**Primer sequence (5′-3′)**	**Reference**
F5Δ*hmuSTUV*	ATGATGGGATCCCCGATGTCGATGCCGATAAA	This study
R5Δ*hmuSTUV*	GATAAATCTGTGGCAGGATGCGTTCATAATGGCTTCCTAA	This study
F5Δ*hmuRSTUV*	ATGATGGGATCCCAATGCGAACTATCAGGGTAATC	This study
R5Δ*hmuRSTUV*	GATAAATCTGTGGCAGCATGTCGGCAATTCTCCATATT	This study
F3Δ*hmuRSTUV*	CTGCCACAGATTTATCTGCGGCAATA	This study
R3Δ*hmuRSTUV*	ATGATGGCGGCCGCGTTGATGCCAGATAGTGTGTATA	This study
qPCR-hmuR-F	GGATGCCAGCATGAGCTATAA	This study
qPCR-hmuR-R	GTCGAGCCATTCATCGGTATT	This study
qPCR-hmuS-F	CCGCTATGAGAACCAACACTTA	This study
qPCR-hmuS-R	GGTCTCTTCGGTCAGTGTAAAG	This study
qPCR-16S-F	AGCCAGCGGACCACATAAAG	Arafah et al., 2009
qPCR-16S-R	AGTTGCAGACTCCAATCCGG	Arafah et al., 2009

### Protein purification

*E. coli* IscR-C92A was anaerobically purified as previously described (Giel et al., 2006; Nesbit et al., 2009). *E. coli* Fur was purified as described in Lee et al. (2003) except that a pET-11a overexpression vector containing *fur* was used (pPK11241). Cultures were grown in LB containing ampicillin (50 μg/mL) at 37°C to an OD_600_ of 0.6 and Fur synthesis was subsequently induced by addition of 400 μM IPTG for 2.5 h at 37°C.

### Electrophoretic mobility shift assays (EMSAs)

DNA fragments containing the intergenic promoter region between *hmuR* and *hmuSTUV* (−143 to +72 bp relative to the *hmuS* start codon), and a control DNA fragment (−280 to −123 bp relative to the *hmuS* start codon), were respectively isolated from pPK12468 and pPK12469 after digestion with *Hind*III and *Bam*HI. These fragments and linearized plasmid (which served as competitor DNA in the EMSAs) were purified with Elutip-d columns (Schleicher and Schuell). IscR-C92A was incubated with DNA fragments (~5–10 nM) for 30 min at 37°C in 40 mM Tris (pH 7.9), 30 mM KCl, 100 μg/mL bovine serum albumin (BSA), and 1 mM DTT. Samples were loaded onto a non-denaturing 6% polyacrylamide gel in 0.5× Tris-borate-EDTA (TBE) buffer and run at 100 V for 90 min. The gel was stained with SYBR Green EMSA nucleic acid gel stain (Molecular Probes) and visualized using a Typhoon FLA 900 imager (GE). These assays (along with other *in vitro* assays performed with IscR-C92A described below) were carried out under aerobic conditions with IscR-C92A that was diluted immediately before use.

Assays performed with *E. coli* Fur were carried out in a similar manner except that Fur (which was pre-equilibrated with 100 μM MnCl_2_ at room temperature for 20 min to form active Mn^2+^-Fur) was incubated with DNA fragments in 20 mM BisTris (pH 7.5), 1 mM MgCl_2_, 40 mM KCl, 100 μM MnCl_2_, 100 mM DTT, 5% glycerol, 100 μg/mL BSA. DNA fragments encompassing the *hmuR* promoter (−100 to −1 bp relative to the *hmuR* transcriptional start site) or the intergenic promoter region between *hmuR* and *hmuSTUV* (−123 to −1 bp relative to the *hmuS* start codon) were PCR amplified from pFU99::p1 and pFU99::p2, respectively, and purified with Elutip-d columns (Schleicher and Schuell).

### DNase I footprinting

The intergenic promoter region between *hmuR* and *hmuSTUV* (−123 to −1 bp relative to the *hmuS* start codon) was isolated from pPK12440 after digestion with HindIII and BamHI. Sequenase version 2.0 (USB Scientific) was used to 3′-end radiolabel the BamHI end of the fragment (top strand) with [α-^32^P]dGTP (Perkin Elmer). The labeled fragment was isolated from a non-denaturing 5% acrylamide gel and purified with Elutip-d columns (Schleicher and Schuell). Footprinting was performed by incubating IscR-C92A with labeled DNA (~5 nM) for 30 min at 37°C in 40 mM Tris (pH 7.9), 30 mM KCl, 100 μg/mL BSA, and 1 mM DTT followed by the addition of 2 μg/mL DNase I (Worthington) for 30 s. The reaction was terminated by the addition of sodium acetate and EDTA to final concentrations of 300 and 20 mM, respectively, and the reaction mix was ethanol precipitated, resuspended in urea loading dye, heated for 60 s at 90°C, and loaded onto a 7 M urea-8% polyacrylamide gel in 0.5× Tris-borate-EDTA (TBE) buffer. An A+G ladder was made by formic acid modification of the radiolabeled DNA, followed by piperidine cleavage (Maxam and Gilbert, 1980). The reaction products were visualized by phosphorimaging.

### *In vitro* transcription assay

The effect of IscR-C92A on σ^70^-dependent promoter activity from candidate control regions was determined by incubating IscR-C92A with 2 nM supercoiled pPK12440 [purified with the QIAfilter Maxi kit (Qiagen)], 0.25 μCi of [α-^32^P]UTP (3,000 μCi/mmol; Perkin Elmer), 20 μM UTP, and 500 μM each of ATP, GTP, and CTP for 30 min at 37°C in 40 mM Tris (pH 7.9), 30 mM KCl, 10 mM MgCl_2_, 100 μg/mL bovine serum albumin (BSA), and 1 mM DTT. Eσ^70^ RNA polymerase (NEB) was added to a final concentration of 50 nM and the reaction was terminated after 5 min by addition of Stop Solution (USB Scientific). Samples were heated for 60 s at 90°C, and loaded onto a 7 M urea-8% polyacrylamide gel in 0.5× Tris-borate-EDTA (TBE) buffer. The reaction products were visualized by phosphorimaging.

### RNA isolation and library preparation

For RNA isolated from samples subjected to iron starvation, *Y. pseudotuberculosis* WT and Δ*iscR* strains were grown in 5 mL of M9 minimal media overnight. Cultures were then diluted to OD_600_ of 0.1 in 20 mL of M9 media treated with Chelex (Bio-Rad) to remove iron and allowed to grow for 8 hrs. The cultures were then diluted again to OD_600_ 0.1 into 20 mL Chelex treated M9 media and allowed to grow for 12 h. Cultures were then diluted to OD_600_ of 0.1 into 20 mL Chelex treated M9 media with either no iron, 5 μM hemin, or 1 mg/L FeSO_4._ Before addition, stock hemin solution was Chelex-treated overnight. After 3 h of growth at 37°C, 5 mL of culture from each condition was pelleted by centrifugation for 5 min at 4,000 rpm.

For all samples, the supernatant was removed and pellets were resuspended in 500 μL of media and treated with 1 mL Bacterial RNA Protect Reagent (Qiagen) according to the manufacturer's protocol. Total RNA was isolated using the RNeasy Mini Kit (Qiagen) per the manufacturer's protocol. Contaminating DNA was removed using the TURBO DNA-free Kit (Life Technologies/Thermo Fischer). rRNA was removed using the RiboZero Magnetic Kit for Gram Negative Bacteria (Illumina). The cDNA library was prepared using the NEB Ultra Directional RNA Library Prep Kit for Illumina. Quality of total RNA, mRNA after rRNA depletion and cDNA libraries were assessed using an Agilent 2000 Bioanalyzer.

### RNA sequencing and analysis

These studies were performed with three biological replicates per condition. RNA-Seq analysis on bacteria that were not starved for iron (**Figure 2A**) was previously published (Miller et al., 2014). For cultures depleted of iron and either left iron depleted or given FeSO_4_ or hemin (**Figures 2B–D**), indexed samples were sequenced using a MiSeq Illumina sequencing platform for 150 bp end reads (UC Davis Genome Center). The full RNA-Seq data set from these iron depleted cultures will be published separately (Wei, Schwiesow, Balderas, and Auerbuch, data not shown). All sequencing data was analyzed and visualized via the CLC Genomics Workbench version 9.5.3 (CLC bio). Reads were mapped to the *Yersinia pseudotuberculosis* genome (IP32953). Differentially regulated genes were identified as those displaying a fold change with an absolute value of 2 or greater. Statistical significance was determined using an EdgeR-like analysis within the RNAseq plugin in CLC workbench with a corrected FDR *post-hoc* test where *p* ≤ 0.01 was deemed significant.

### Quantitative reverse transcriptase PCR (qRT-PCR) analysis

After harvesting total RNA from wild-type or Δ*iscR* strains cultured under the indicated conditions via the procedure described above, genomic DNA was removed via the TURBO-DNA-free kit (Life Technologies/Thermo Fisher). cDNA was generated for each sample by using the M-MLV Reverse Transcriptase (Invitrogen) according to the manufacturer's instructions, as we previously described (Miller et al., 2014). Each 20 μl qRT-PCR assay contained 5 μl of 1:10 diluted cDNA sample, 10 μl of Power CYBR Green PCR master mix (Thermo Fisher Scientific), and primers (Table 2) with optimized concentrations. The expression levels of each target gene were normalized to that of 16S rRNA present in each sample, and calculated by the ΔΔCt method. Three independent biological replicates were harvested for each tested condition. For each target transcript, significant differential expression between different bacterial strains were defined by *p*-value < 0.05 of one-way analysis of variance (one-way ANOVA).

### Hemin growth assays

The effect of addition of hemin on bacterial growth was assayed in liquid cultures similar to Thompson et al. (1999). *Y. pseudotuberculosis* was grown as described in the RNA-Seq section above. OD_600_ was measured every hour for 9 h. Data is representative of three independent experiments.

### Whole blood assay

Strains were grown overnight in 2xYT at 26°C and subsequently standardized to an OD_600_ of 0.2. Standardized cultures were centrifuged at 3,500 rpm for 3 min and the supernatants discarded. Bacterial pellets were resuspended in 2 mL of whole sheep's blood in sodium heparin (Hemostat, Inc.) and CFU determined via serial dilution and plating at hour 0. Samples were then placed in a rollerdrum at 37°C and CFU determined at hours 2, 4, 6, and 8. Data is representative of three independent experiments. We observed blood batch to batch variability in terms of the dynamics of bacterial survival; however, the difference between WT and Δ*iscR* survival were observed for all batches of blood.

### Serum growth assay

Non-heat inactivated sheep serum did not exhibit killing of *E. coli* K12 or *Yersinia* (data not shown), so bovine serum was used. *Y. pseudotuberculosis* or *E. coli* K12 was grown in 5 mL of M9 minimal media overnight. In the morning, cultures were diluted to OD_600_ 0.05 in non-heat treated bovine serum (innovative technologies), or bovine serum that had been heat-killed for 30 min at 60°C. OD_600_ was measured every hour for 5 h.

## Results

### Changes in iron availability control expression of *hmuR* and *hmuSTUV* in distinct ways

**Figure 1:**
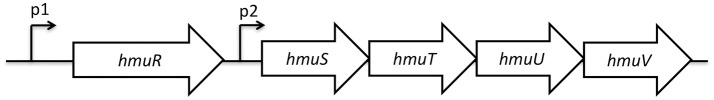
Genetic structure of the *hmuRSTUV* genetic locus. The promoter upstream of *hmuR* is denoted as p1, while the intergenic promoter between *hmuR* and *hmuS* is denoted at p2.

**Figure 2:**
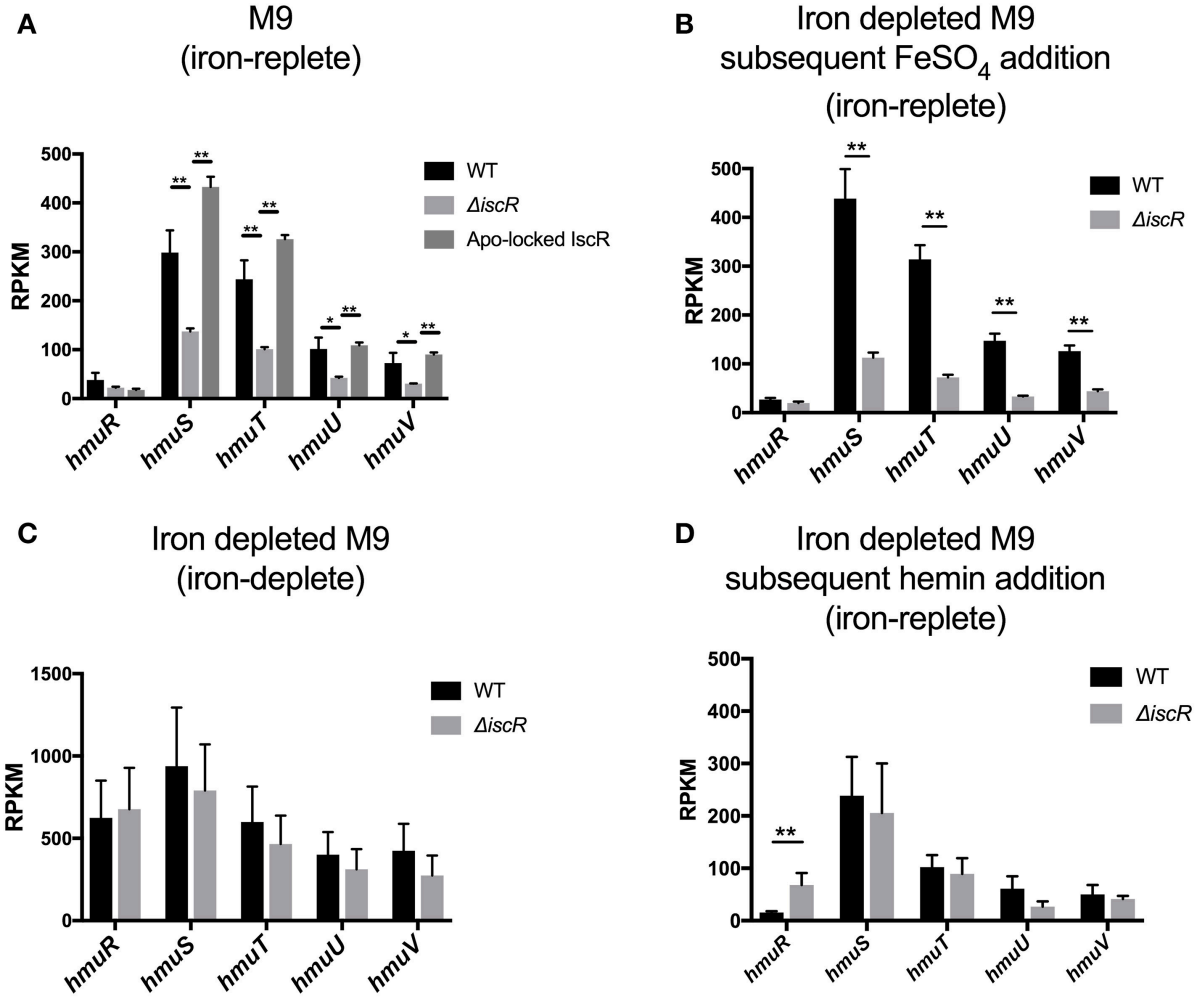
Expression of the *hmuRSTUV* locus genes under varying iron conditions measured by RNA-Seq. Reads per Kilobase per transcript per Million mapped reads (RPKM) of WT and Δ*iscR* strains grown in **(A)** M9 containing FeSO_4_, or iron starved in Chelex-treated M9 and **(B)** grown in FeSO_4_ for 3 h, **(C)** no iron source added back, or **(D)** grown in 5 mM hemin for 3 h. Details on iron starvation can be found in the materials and methods. ^*^p ≤ 0.05, ^**^p ≤ 0.001 (EdgeR with a corrected FDR *post-hoc* test).

**Figure 3:**
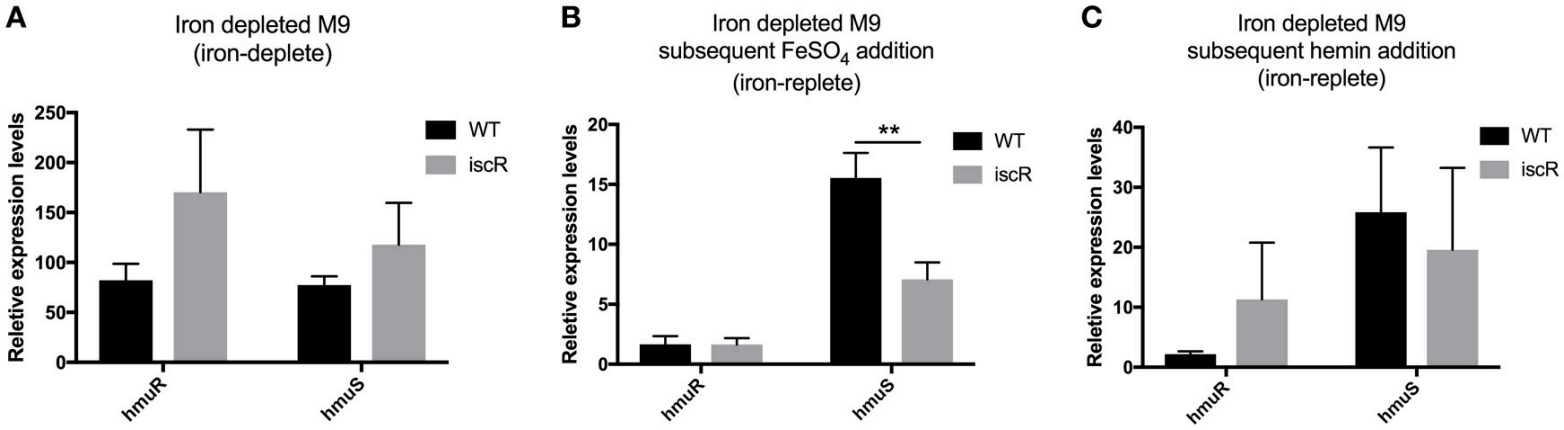
Expression of the *hmuRSTUV* locus genes under varying iron conditions measured by qPCR. Quantitative reverse transcriptase PCR (qRT-PCR) was performed to measure *hmuR* and *hmuS* relative expression levels in WT and Δ*iscR* strains iron starved and grown as in Figures 2B–D, **(A)** in iron depleted M9, **(B)** with FeSO_4_ for 3 h, and **(C)** with 5 mM hemin for 3 h. Relative expression levels of *hmuR* and *hmuS* were normalized to that of 16S rRNA in the same sample and calculated by the ΔΔCt method. Details on iron starvation can be found in the materials and methods. ^**^p ≤ 0.01 (One-way ANOVA).

The genetic structure of the *hmuRSTUV* hemin uptake locus has been characterized in *Yersinia pestis* (Thompson et al., 1999). This locus contains two promoter regions: one upstream of *hmuR* (p1), and one in the intergenic region between *hmuR* and *hmuS* (p2; Figure 1) (Thompson et al., 1999). Previous work suggested that p1, but not p2, promoter activity was increased by iron limitation through a Fur-dependent mechanism (Thompson et al., 1999). Here we extend this study by using RNA-seq analysis in the related pathogen *Y. pseudotuberculosis* to measure expression of *hmuRSTUV* under conditions when cells had been depleted of iron or when inorganic iron or hemin were added back as iron sources after a period of iron starvation (Figures 2B–D, Supplementary Dataset). In the presence of added inorganic iron (1.0 mg/L FeSO_4_; iron replete conditions), levels of *hmuR* mRNA (RPKM values) were significantly less than those of *hmuSTUV* (Figure 2B). This pattern of RNA expression was similar to what we previously observed (Miller et al., 2014) when *Yersinia* was grown in standard M9 minimal media with no period of iron starvation (Figure 2A). Surprisingly, addition of hemin to iron deprived cells actually decreased expression of *hmuSTUV* compared to cells with added inorganic iron (Figure 2D). In contrast, in the absence of added iron following a period of iron starvation (iron depletion), *hmuR* mRNA levels were upregulated (Figure 2C), consistent with previous reports in *Y. pestis* (Thompson et al., 1999). Furthermore, the RPKM values now showed that *hmuR* expression was nearly equivalent to *hmuSTUV* (Figure 2C). This finding was also validated by qPCR (Figures 3A,B). In addition, reads spanning the intergenic region between *hmuR* and *hmuS* were detected by RNA-Seq, and a transcript containing *hmuR* and *hmuS* was detected by RT-PCR under iron depleted conditions, indicating some contiguous transcription under these conditions (data not shown). This may be a result of read-through transcription from the *hmuR* promoter in the absence of iron stemming from Fur de-repression (see below). In summary these data suggest complex transcriptional regulation of the *hmuRSTUV* locus in *Y. pseudotuberculosis*.

### Fur directly binds to the promoter upstream of *hmuR*, but not to the intergenic region between *hmuR* and *hmuS*

**Figure 4:**
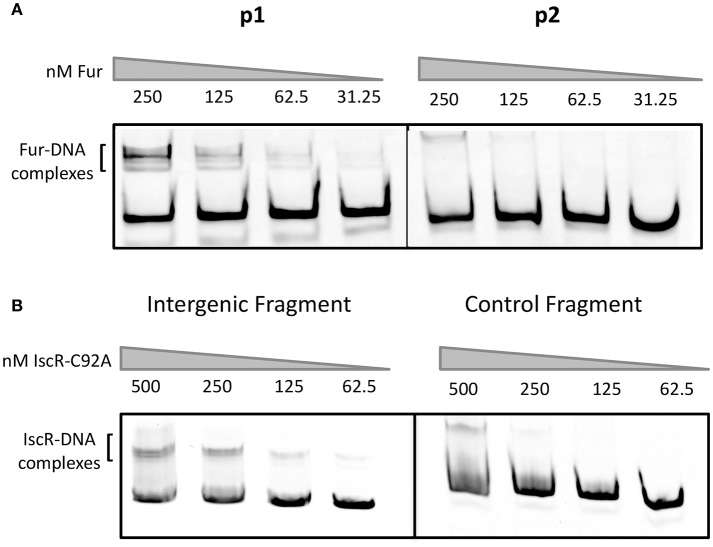
*E. coli* Fur binds to the promoter upstream of *hmuR* and *E. coli* IscR-C92A binds to the intergenic region between *hmuR* and *hmuSTUV*. **(A)** Electrophoretic mobility shift assays (EMSAs) using DNA from the promoter regions shown in Figure 1. Concentrations of Fur protein used in the gel shift assays are denoted above the gel lanes. **(B)** EMSAs using DNA from the intergenic region between *hmuR* and *hmuSTUV* or control DNA within the *hmuR* coding region. Concentrations of Apo-locked IscR-C92A protein used in the gel shift assays are denoted above the gel lanes.

Previous work in *Y. pestis* suggested that Fur regulates the promoter upstream of *hmuR* and that *hmuSTUV* genes were expressed from a weak, constitutive promoter in the intergenic region between *hmuR* and *hmuS* (Thompson et al., 1999). However, Fur binding to these regions was not tested biochemically. To determine if Fur directly binds to the *hmuR* promoter and/or the intergenic promoter between *hmuR* and *hmuS* in *Y. pseudotuberculosis*, we performed gel shift assays using purified *E. coli* Fur protein. When incubated with increasing amounts of Fur protein, a shift was observed for the promoter region upstream of *hmuR* at 31.25 nM protein, while no binding was detectable for the intergenic region between *hmuR* and *hmuS* until the protein concentration was increased 8-fold (Figure 4A). These data suggest that Fur directly regulates the *hmuRSTUV* heme uptake locus through direct binding to the promoter upstream of *hmuR*, and not through binding the intergenic region between *hmuR* and *hmuS*. Thus, the increased *hmuRSTUV* RNA levels observed under iron depletion conditions (Figure 2) are likely explained by the loss of Fur binding to the *hmuR* promoter.

### IscR plays a role in regulation of *hmuSTUV*

Previous analysis of RNA-Seq data from our group suggested that IscR also plays a role in the regulation of the *hmuRSTUV* hemin uptake system (Miller et al., 2014). While *hmuR* levels were not altered by deletion of *iscR* as measured by RNA-Seq and qPCR, *hmuSTUV* levels were significantly decreased in the Δ*iscR* mutant compared to WT and an apo-locked mutant variant strain under iron replete conditions (Figure 2A). This pattern of expression is consistent with IscR activation of *hmuSTUV* by binding to an IscR type II motif since both WT and apo-locked mutant were able to increase expression. As expected for a type II motif that binds either apo-IscR or holo-IscR, the requirement for IscR was also observed when cultures were starved of iron for several days and then given an inorganic iron source (Figures 2B, 3B). However, the presence of hemin appeared to disable the increased expression by IscR since RNA levels were not further enhanced when IscR was present (Figures 2D, 3C). Moreover, the difference in *hmuSTUV* RNA levels between the WT and Δ*iscR* strains was eliminated when the bacteria remained starved for iron (Figures 2C, 3A). This is in part likely a result of increased transcription from the *hmuR* promoter stemming from Fur de-repression, which apparently transcribes through *hmuSTUV* under these conditions. Collectively, these data suggest that the source of iron plays a critical role in determining the relative amount and ratios of *hmuR to hmuSTUV* mRNAs and that IscR and Fur play distinct roles in *hmu* regulation.

### Apo-IscR directly binds to the intergenic region between *hmuR* and *hmuS*

**Figure 5:**
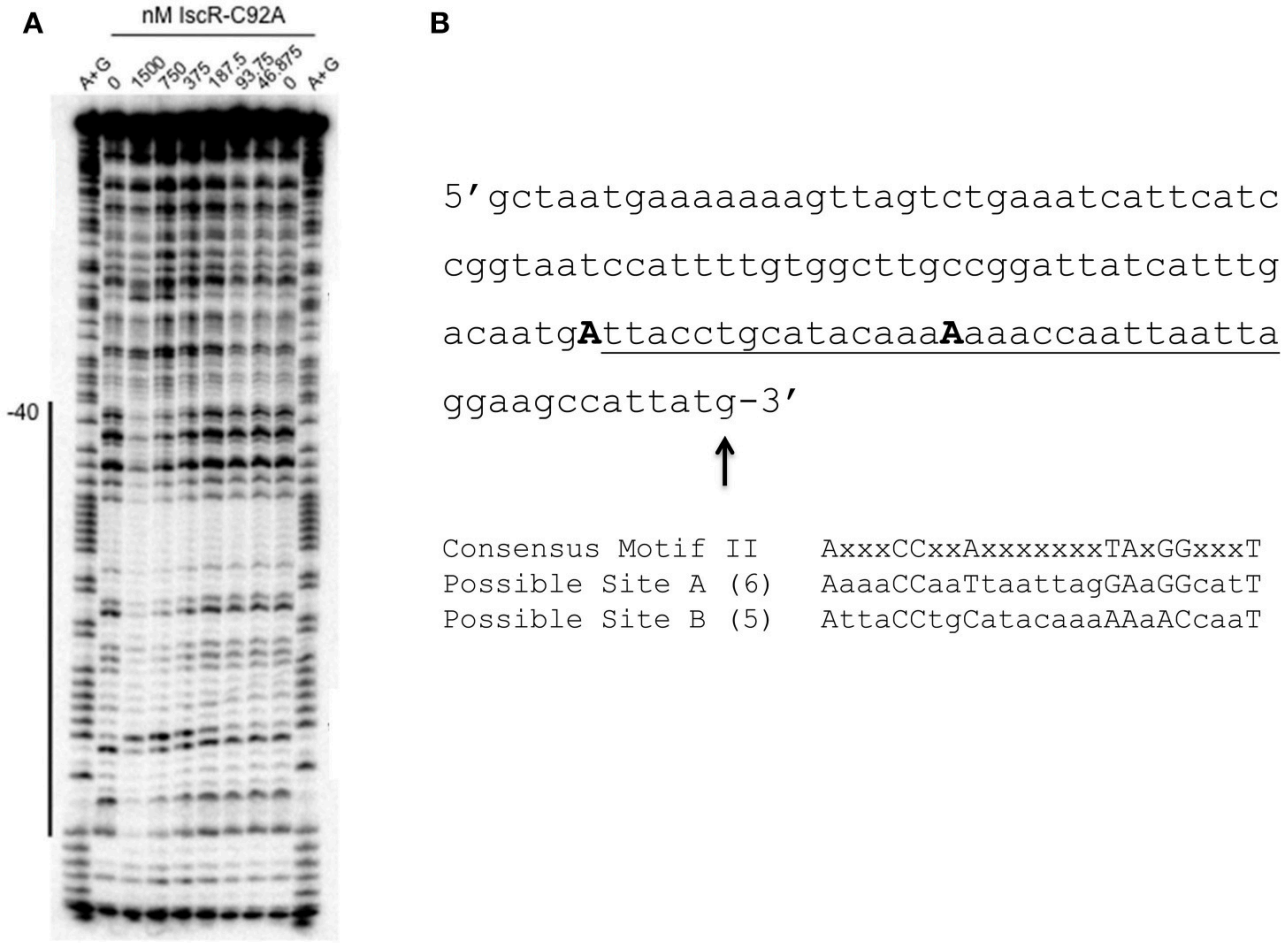
*E. coli* IscR-C92A DNase I footprinting reveals IscR Motif II binding sites in the intergenic region between *hmuR* and *hmuS*. **(A)** DNase I footprinting of IscR-C92A binding to the intergenic region between *hmuR* and *hmuS*. The labeled DNA is the top strand from −123 to−1 relative to the *hmuS* translational start site. The region protected by IscR-C92A is marked by a black line and −40 denotes the position from the *hmuS* translational start site. **(B)** Sequence of the intergenic region between *hmuR* and *hmuS*. The region of protection by IscR-C92A is underlined. Possible Motif II Sites are marked below the sequence and the number of nucleotides contained in each sequence shown to be important for IscR binding are denoted. The first residue of each possible motif are capitalized and bolded.

We asked if IscR could directly bind to the intergenic region between *hmuR* and *hmuS* (Figure 4B). *E. coli* IscR has been shown to complement a *Y. pseudotuberculosis* Δ*iscR* mutant and is 100% identical in its DNA binding domain to *Yersinia* IscR (Miller et al., 2014). We used *E. coli* IscR-C92A protein that has one Fe-S cluster coordinating cysteine mutated to assay DNA binding because it is in the apo-locked conformation, and binds type II sites in the absence of the [2Fe-2S] cluster allowing DNA binding to be assayed under normal laboratory conditions. We found that purified IscR-C92A was bound toward the 3′ end of the p2 fragment used in our Fur binding studies (Figure 4A; data not shown). To characterize the IscR binding site further, we used a larger fragment containing the intergenic region between *hmuR* and *hmuS* and a control fragment containing a region within the 3′ end of the *hmuR* coding region. When incubated with increasing concentrations of IscR-C92A protein, a shift was observed for the intergenic region DNA at 62.5 nM protein, while a shift for the control DNA fragment was not observed until 250 nM protein was used (Figure 4B). To further define the IscR binding site, we performed DNase footprinting assays with *E. coli* IscR-C92A. A region of protection was observed from −40 to −1 nucleotides upstream of the translational start site of *hmuS* (Figure 5A). From this area of protection, we identified two potential IscR type II motif sites, termed site A and site B (Figure 5B). Comparison of these binding sites with the consensus type II motif shows that they contain six and five of the nine bases in the consensus binding motif, respectively (Figure 5B) (Giel et al., 2006). These data suggest that IscR directly binds to the intergenic region between *hmuR* and *hmuS* through one or two type II motifs.

### IscR is not sufficient to activate transcription from the intergenic promoter between *hmuR* and *hmuS*

**Figure 6:**
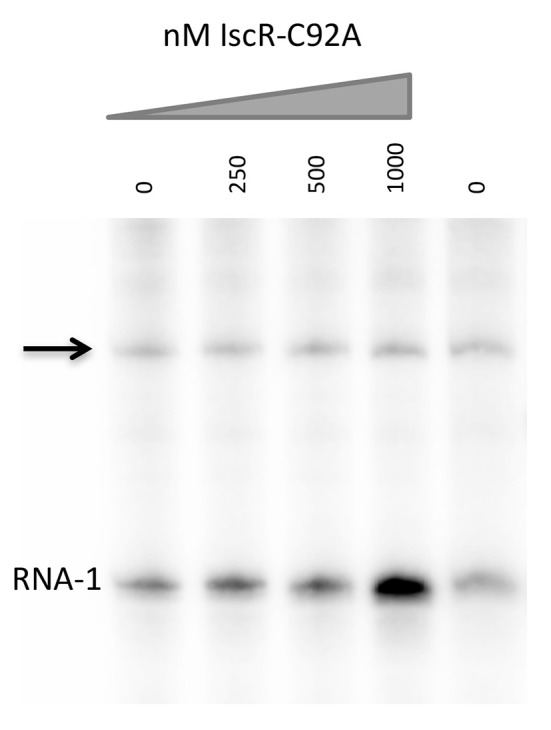
*E. coli* IscR-C92A cannot activate transcription from the intergenic promoter between *hmuR* and *hmuS*. *In vitro* transcription reactions contain plasmids harboring the intergenic promoter between *hmuR* and *hmuS*, Eσ^70^ RNA polymerase, and increasing concentrations of IscR-C92A protein. Transcripts from the intergenic promoter are marked with an arrow and transcripts from the control RNA-1 promoter are indicated.

We asked whether apo-IscR binding to the intergenic region between *hmuR* and *hmuS* directly increased *hmuSTUV* transcription as predicted from the *in vivo* RNA analysis. We performed *in vitro* transcription assays with E-σ70 RNA Polymerase to determine if IscR could drive transcription from the intergenic promoter. We observed no increase in transcript levels over RNA polymerase alone when IscR-C92A was added to the assay (Figure 6). Given these data and the fact that IscR directly binds to this region at one or two putative type II motifs, we hypothesize that IscR functions with another transcriptional regulator to affect transcription of *hmuSTUV*.

### IscR is required for survival in whole blood

**Figure 7:**
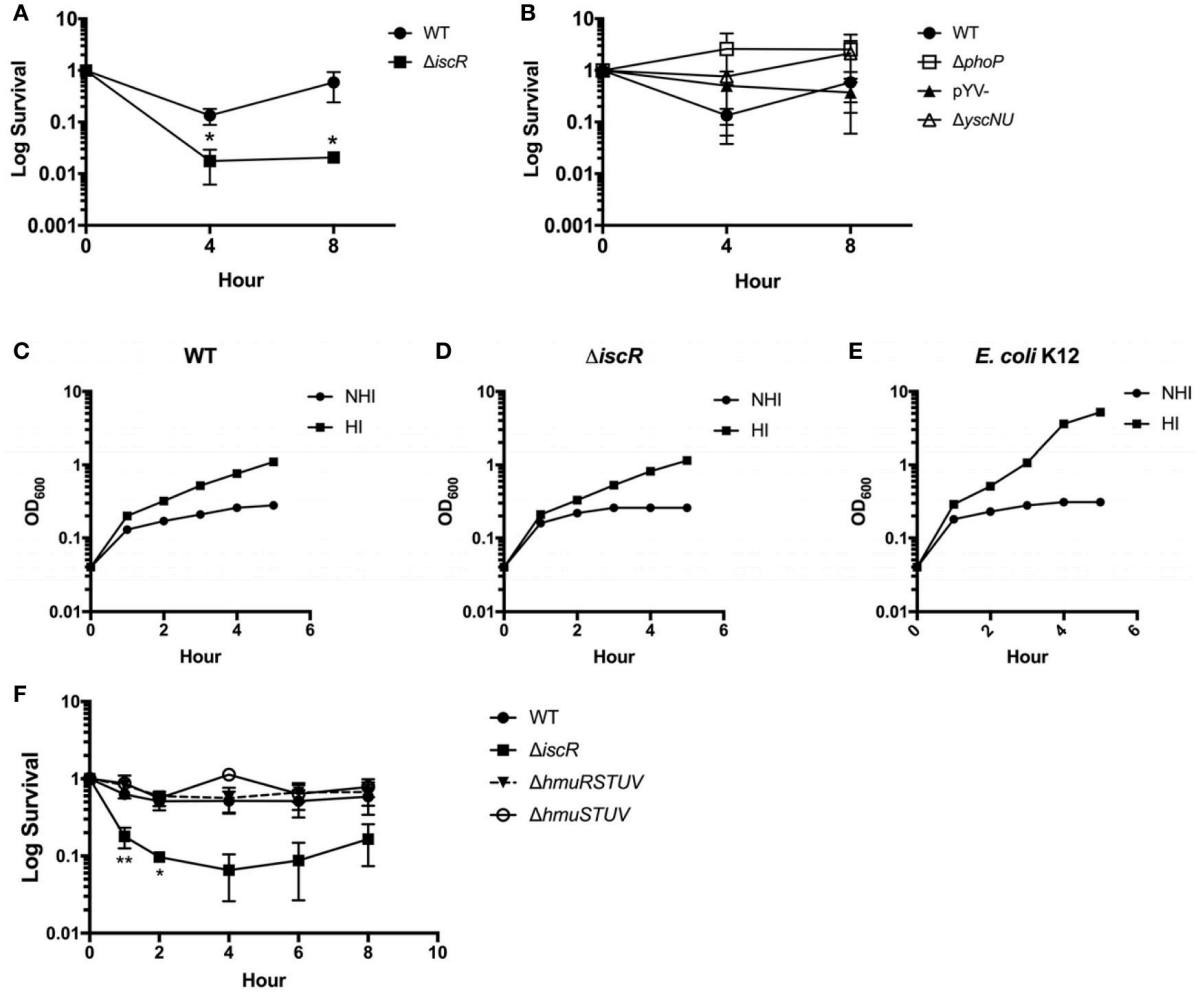
An Δ*iscR* mutant has a survival defect in sheep's whole blood. **(A)** WT and Δ*iscR* log survival in sheep's whole blood. **(B)** WT, Δ*phoP*, pYV-, and Δ*yscNU* log survival in sheep's whole blood. Log survival is calculated as the CFU/mL bacteria at 4 or 8 h divided by the CFU/mL bacteria at hour 0 as enumerated by plating for CFU. Data are from three biological replicates.^*^p ≤ 0.05, unpaired student *t*-test compared to WT. **(C–E)** *E. coli* K12 and *Y. pseudotuberculosis* WT and Δ*iscR* were incubated in heat-inactivated (HI) and non heat-inactivated (NHI) bovine serum and bacterial growth monitored by measuring optical density. **(F)** WT, Δ*iscR*, Δ*hmuRSTUV*, and Δ*hmuSTUV* log survival in sheep's whole blood. Log survival is calculated as the CFU/mL bacteria at 1, 2, 4, 6, or 8 h divided by the CFU/mL bacteria at hour 0 as enumerated by plating for CFU. Data are from two to three biological replicates (depending on time point). ^*^p < 0.05 and ^**^ <0.01, unpaired Student *t*-test compared to WT.

Our previous work showed that *Y. pseudotuberculosis* lacking *iscR* is severely attenuated in disseminated infection following oral inoculation of mice (Miller et al., 2014). As IscR regulates expression of the Hmu heme uptake locus, we assessed whether IscR was important for *Yersinia* survival in the heme rich environment of blood by measuring the ability of *Y. pseudotuberculosis* to survive in whole sheep's blood. Even in the absence of starving the bacteria of iron, colony forming units (CFU) of the Δ*iscR* mutant were decreased by 1-2-logs compared to the WT strain at 4 and 8 h (Figure 7A). This suggests that IscR plays a role in the survival of *Y. pseudotuberculosis* in blood.

We previously characterized IscR as important for expression of the T3SS through the master regulator LcrF in *Y. pseudotuberculosis* (Miller et al., 2014). The *Yersinia* T3SS, encoded on the pYV virulence plasmid, is important for the evasion of phagocytic cells, which may be present in our *ex vivo* blood model (Raymond et al., 2013). Additionally, the pYV-encoded protein YadA, which is controlled by LcrF, has been shown to be important in the evasion of complement in *Yersinia enterocolitica* through exploitation of C3 and iC3b to sequester large amounts of Factor H (Skurnik and Toivanen, 1992; Schindler et al., 2012). Therefore, we assessed the requirement for IscR-mediated T3SS and YadA control in the survival of *Y. pseudotuberculosis* in blood. We observed that neither a pYV^−^ strain of *Y. pseudotuberculosis* that lacks the T3SS and YadA, nor a Δ*yscNU* mutant that lacks critical T3SS basal body components, display survival defects in whole blood compared to WT (Figure 7B). These data suggest that IscR-mediated T3SS and YadA regulation is not important for survival of *Y. pseudotuberculosis* in our blood model. Furthermore, the Δ*iscR* mutant and WT strains survived equally well in heat inactivated and non-heat inactivated bovine serum (Figures 7C–E), ruling out the involvement of complement in killing of the Δ*iscR* mutant. These data indicate that either the cells or clotting factors present in whole blood but not in serum are responsible for killing the Δ*iscR* mutant. The response regulator PhoP is required for *Y. pseudotuberculosis* to replicate in macrophages (Grabenstein et al., 2004). We found that a Δ*phoP* mutant does not display a survival defect in blood (Figure 7B), suggesting that the inability of the Δ*iscR* mutant to survive in blood is not due to an inability to replicate in macrophages.

### The *hmurSTUV* locus contributes to *Y. pseudotuberculosis* growth in the presence of heme as the sole iron source, but is dispensable for growth on non-heme iron

**Figure 8:**
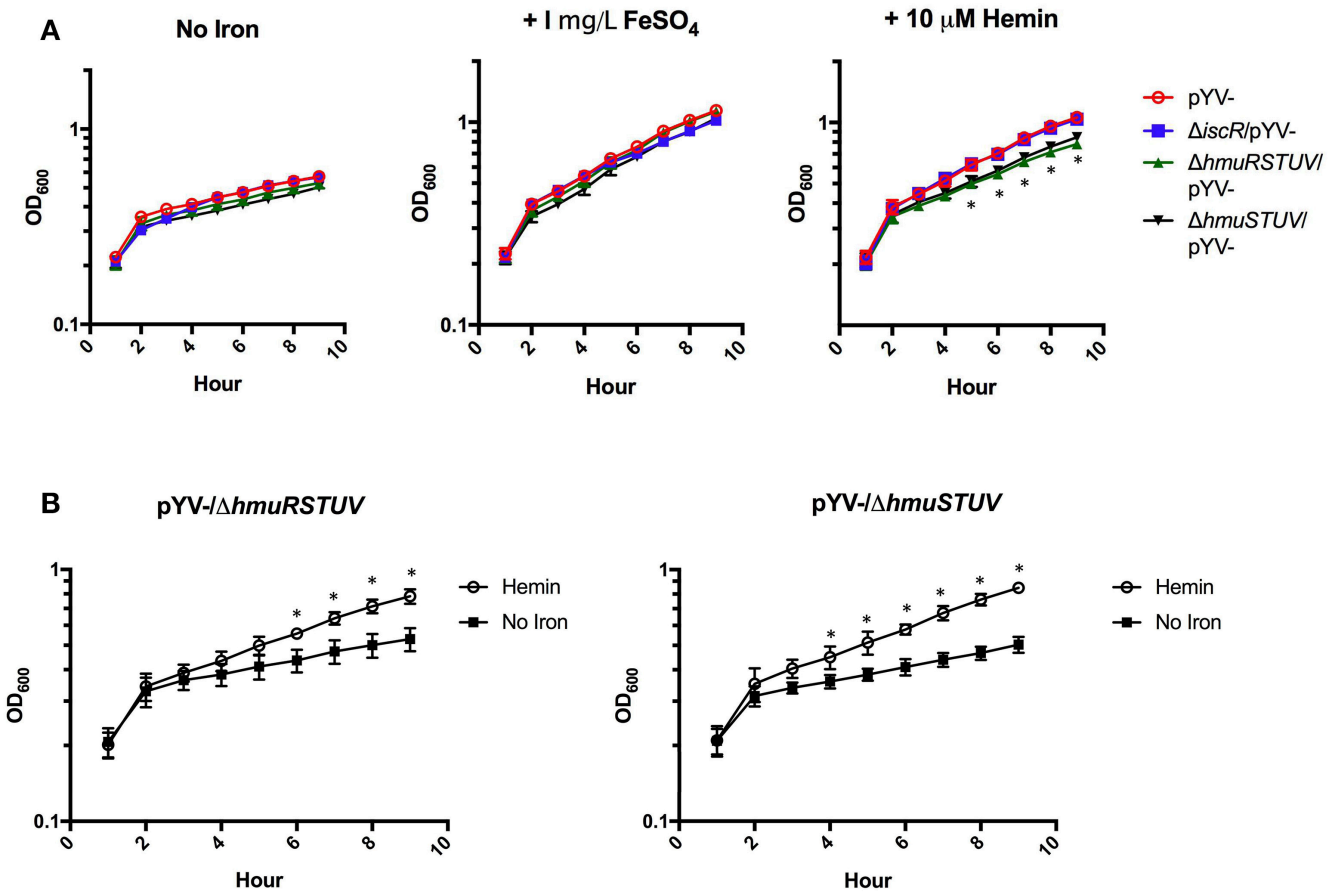
The Hmu heme uptake locus contributes to *Y. pseudotuberculosis* growth on hemin but is dispensable for growth on a non-heme iron source. Iron starved *Y. pseudotuberculosis* were transferred to Chelex-treated M9-Fe media alone or supplemented with 1 mg/L FeSO_4_ or 10 μM Hemin. Cultures were grown at 37°C for 9 h and optical density at 600 nm was taken every hour. **(A)** pYV^−^, pYV^−^/Δ*iscR*, pYV^−^/Δ*hmuRSTUV*, and pYV^−^/Δ*hmuSTUV* are shown for each iron condition. **(B)** ΔpYV^−^/Δ*hmuRSTUV* and pYV^−^/Δ*hmuSTUV* are shown with and without hemin. Data are representative of three independent experiments. ^*^p ≤ 0.05 for Δ*hmuSTUV*/pYV^−^ and Δ*hmuRSTUV*/pYV^−^, One-way ANOVA.

*HmuRSTUV* have been shown to be necessary for the utilization of heme under iron depleted conditions in *Y. pestis* (Hornung et al., 1996; Thompson et al., 1999). Therefore, we examined whether IscR and the Hmu locus played a role in heme utilization in *Y. pseudotuberculosis*. We assessed the ability of pYV^−^, Δ*iscR*/pYV^−^, Δ*hmuRSTUV*/pYV^−^, and Δ*hmuSTUV*/pYV^−^ mutant strains to grow in the presence of hemin or inorganic iron as the sole iron sources, following several days of iron starvation to deplete iron stores. We chose to use the pYV^−^ background because WT *Y. pseudotuberculosis* undergoes growth restriction in M9 minimal media at 37°C due to T3SS activity, while a strain lacking pYV does not. While all strains could utilize FeSO_4_ as an iron source, Δ*hmuRSTUV*/pYV^−^ and Δ*hmuSTUV*/pYV^−^ were defective in growth on hemin compared to the pYV^−^ parental strain (Figure 8A), suggesting that the Hmu system promotes *Y. pseudotuberculosis* growth in heme as the sole iron source. Surprisingly, growth of the Δ*iscR*/pYV^−^ strain was indistinguishable from the pYV^−^ parental strain, indicating that *hmu* expression levels in the Δ*iscR*/pYV^−^ mutant were sufficient to sustain *Yersinia* growth on the amount of hemin provided. Indeed, there was no significant difference in *hmuSTUV* RPKM levels between the WT and Δ*iscR* strains 3 h after hemin addition (Figures 2D, 3C). Interestingly, *Yersinia* could still utilize the iron in the hemin provided in our experiment in the absence of the Hmu pathway, as the Δ*hmuRSTUV*/pYV^−^ and Δ*hmuSTUV*/pYV^−^ strains still had residual growth in heme compared to no iron source added (Figure 8B). Our hemin stocks had to be Chelex-treated to remove free iron prior to start of the growth curve in order to observe a significant difference in growth between the parental strain and the *hmu* mutants (Figure 8A, unpublished observations). This suggests that residual iron-stimulated growth may occur from breakdown of heme followed by iron uptake by non-heme uptake systems under our growth assay conditions.

While the Hmu locus contributes to *Yersinia* growth on M9 plus hemin following iron starvation, the Δ*hmuRSTUV* and Δ*hmuSTUV* mutants did not display a defect in our *ex vivo* blood model, rich in heme (Figure 7F). This finding may stem from the lack of iron starvation of the mutants before the survival assays in whole blood were performed. Indeed, to observe the Δ*hmuRSTUV and* Δ*hmuSTUV* growth defect in M9 media plus hemin, the bacteria had to be iron-starved to deplete iron stores prior to hemin exposure (data not shown). These data also indicate that the survival defect of the Δ*iscR* mutant in blood does not stem from diminished heme utilization under our experimental conditions.

## Discussion

In this study, we present evidence that IscR plays distinct roles in regulating heme utilization and survival in blood in the bacterial pathogen *Yersinia pseudotuberculosis*. DNA binding analysis revealed IscR binding to the intergenic region between *hmuR* and *hmuS* at one or two putative type II motifs. We also observed that an Δ*iscR* mutant cannot survive as well as the WT strain in whole blood. We propose that the severe virulence attenuation of the Δ*iscR* mutant seen in a mouse oral infection model may, in part, be due to a defect in blood survival that is independent of the T3SS, complement, and the ability of *Yersinia* to replicate in macrophages.

DNase footprinting revealed two putative IscR type II binding motifs in the intergenic region between *hmuR* and *hmuS* that contain five or six of the nine bases found to be important for IscR binding (Nesbit et al., 2009). This and data from the *hmu* transcript analysis using an apo-locked IscR allele suggested that IscR in either the holo- or apo- form may drive transcription from the intergenic promoter between *hmuR* and *hmuS*. However, we found that IscR alone cannot itself drive transcription from this promoter, suggesting that IscR either antagonizes a negative regulator or requires another positive regulator to drive *hmuSTUV* expression. This mode of regulation is not unlike IscR regulation of the *sodA* promoter in *E. coli*. The *sodA* promoter is also unresponsive to IscR levels in *in vitro* transcription assays. In this case IscR might act by competing with the known repressor of *sodA*, Fur (Giel et al., 2006; Beauchene et al., 2015) (unpublished data). However, the lack of Fur binding to the intergenic promoter between *hmuR* and *hmuS* suggests that a transcriptional regulator distinct from Fur acts with IscR at the *hmuS* promoter.

Our data suggest that Fur represses *hmuRSTUV* expression by binding to the *hmuR* promoter under iron replete conditions. We propose that when iron becomes limiting, Fur no longer represses transcription from the *hmuR* promoter, enabling maximal transcription of the entire *hmuRSTUV* operon and the potential for heme utilization from the environment. In contrast, when iron is available the *hmuR* promoter is repressed by Fur, but the intergenic promoter can mediate IscR-dependent, moderate expression of *hmuSTUV*. We propose that this dual regulation allows sequestration of heme under iron starvation conditions, while maintaining sufficient machinery (HmuTUV ABC transporter and HmuS heme oxygenase) to remove heme in the periplasm and cytosol in heme and iron-rich environments. Another heme acquisition system, HasRADEB, has been described in *Y. pestis* (Rossi et al., 2001). However, the authors could not find a role for the Has system in *Y. pestis* grown on heme under the conditions they tested. In addition, it is not clear that the *hasRADEB* gene homologs in *Y. pseudotuberculosis* are expressed, as there was no significant difference in *hasRADEB* RPKM levels in our WT strain grown in Chelex treated media with no added iron source compared to media containing FeSO_4_ or hemin (data not shown). It is possible that another, as yet undiscovered, heme uptake system exists in *Y. pseudotuberculosis*. Such a system, if active under our experimental conditions, could explain the ability of the Δ*hmuRSTUV* mutant to grow to a significantly higher optical density in hemin compared to our low iron condition. Alternatively, it is possible that this discrepancy is due to heme degradation in the media and *Yersinia* uptake of the released iron by non-heme iron uptake systems.

We predicted that a Δ*iscR* mutant might be defective in heme uptake. However, while Δ*hmuRSTUV* and Δ*hmuSTUV* mutants had a defect in growth on hemin as the sole iron source, a Δ*iscR* mutant did not. This was unexpected given that our genetic and biochemical data show that IscR is involved in *hmuSTUV* expression. Surprisingly, we did not observe lower *hmuSTUV* expression in the Δ*iscR* strain compared to WT during growth on heme as the sole iron source. It is possible that the Δ*iscR* mutant during growth in the presence of heme has altered expression or activity of transcriptional regulator(s) distinct from Fur and IscR that control *hmu* expression. Interestingly, previous work showed that inorganic iron, but not hemin, caused a decrease in p1 promoter activity in *Y. pestis* (Thompson et al., 1999). Collectively, these data suggest that *Yersinia* IscR and Fur, and possibly other regulators, synergize to control gene expression in response to changes in iron source and availability.

*Y. pseudotuberculosis* carries a small ORF called *hemP* that is directly upstream of, and co-transcribed with, *hmuR* (data not shown). It was recently shown that *Burkholderia multivorans* HemP binds heme, binds to the *hmuR* promoter at a GC-rich inverted repeat region, and acts as a transcriptional activator stimulating the *hmuR* promoter in the absence of Fur repression (Sato et al., 2017). It is possible that in *Yersinia* HemP contributes to *hmu* regulation, as has been shown in *Burkholderia* (Sato et al., 2017). However, *Y. pseudotuberculosis hemP* is truncated compared to *Y. enterocolitica* and *B. multivorans hemP*, and an *in silico* search failed to identify any GC-rich inverted repeats in the *Y. pseudotuberculosis hmuR* promoter or the intergenic promoter between *hmuR* and *hmuS* (data not shown). Future work will determine whether *Yersinia* HemP is a transcriptional regular and if it synergizes with Fur and IscR to control heme utilization.

We performed survival curves using sheep's whole blood, a heme-rich medium. The Δ*iscR* mutant was defective in blood survival and this defect was independent of Hmu-mediated hemin utilization under our experimental conditions. Additionally, we ruled out any contribution of IscR control of the T3SS to survival in blood, as two different T3SS mutants did not display blood survival defects. Furthermore, a mutant lacking *phoP* was not defective in survival in blood, indicating that growth inside blood-borne leukocytes is not essential for *Y. pseudotuberculosis* survival in blood. Lastly, enhanced complement killing of the Δ*iscR* mutant did not underlie its survival defect, as YadA (encoded on pYV) was not required for full survival in blood and the WT and Δ*iscR* strains survived equally well in serum. While the cause of the Δ*iscR* blood survival defect remains unclear, it is likely to contribute to the severe attenuation of the *Y. pseudotuberculosis* Δ*iscR* mutant in disseminated infection (Miller et al., 2014).

In summary, we provide evidence of regulation of the *Y. pseudotuberculosis hmuRSTUV* locus by both Fur and IscR. We show that Fur directly binds to and represses the promoter upstream of *hmuR* whereas IscR binds to the intergenic region between *hmuR* and *hmuS*. However, our data suggest that other regulators contribute to Hmu expression. The fact that the *hmu* system is controlled by at least three different transcriptional regulators may enable control of heme uptake and detoxification in response to several environmental signals. Additionally, we suggest that IscR is important for *Yersinia* survival in blood, contributing to the strong impact of IscR on *Yersinia* virulence.

## Author contributions

VA, LS, HM, and PK designed the study. LS, EM, DB, and NH performed the experiments. LS and YW performed data analysis. LS, VA, and PK wrote the paper.

### Conflict of interest statement

The authors declare that the research was conducted in the absence of any commercial or financial relationships that could be construed as a potential conflict of interest.

## Supplementary material

Supplementary Table 1 RNA-Seq data for *hmu* operon and control genes.
